# Effects of *CYP2C19* Loss-of-Function Variants on the Eradication of *H. pylori* Infection in Patients Treated with Proton Pump Inhibitor-Based Triple Therapy Regimens: A Meta-Analysis of Randomized Clinical Trials

**DOI:** 10.1371/journal.pone.0062162

**Published:** 2013-04-30

**Authors:** Hui-Lin Tang, Yan Li, Yong-Fang Hu, Hong-Guang Xie, Suo-Di Zhai

**Affiliations:** 1 Department of Pharmacy, Peking University Therapeutic Drug Monitoring and Clinical Toxicology Center, Peking University Third Hospital, Beijing, China; 2 General Clinical Research Center and Division of Clinical Pharmacology, Nanjing Medical University Nanjing First Hospital, Nanjing, China; 3 Department of Pharmacology, Nanjing Medical University School of Pharmacy, Nanjing, Jiangsu, China; Charité, Campus Benjamin Franklin, Germany

## Abstract

**Background:**

There are inconsistent conclusions about whether *CYP2C19* variants could affect *H. pylori* eradication rate in patients treated with the proton pump inhibitor (PPI)-based therapy. We therefore performed a meta-analysis of randomized clinical trials (RCTs) to re-evaluate the impact of *CYP2C19* variants on PPI-based triple therapy for the above indication.

**Methods:**

All relevant RCTs in the PubMed, Cochrane Library, EMBASE, Web of Science and two Chinese databases (up to February 2013) were systematically searched, and a pooled analysis was performed with the odds ratio (OR) and 95% confidence interval (CI) by the STATA software.

**Results:**

Sixteen RCT datasets derived from 3680 patients were included. There was no significant heterogeneity across the data available in this meta-analysis. There were significant differences in that rate between homozygous (HomEMs) and heterozygous (HetEMs) extensive metabolizers (OR 0.724; 95% CI 0.594–0.881), between HomEMs and poor metabolizers (PM) (OR 0.507; 95%CI 0.379–0.679), or between HetEMs and PMs (OR 0.688; 95%CI 0.515–0.920), regardless of the PPI being taken. Furthermore, sub-analysis of individual PPIs was carried out to explore the difference across all the PPIs used. A significantly low rate was seen in HomEMs vs. HetEMs taking either omeprazole (OR 0.329; 95%CI 0.195–0.553) or lansoprazole (OR 0.692; 95%CI 0.485–0.988), and also in HomEMs vs. PMs for omeprazole (OR 0.232; 95%CI 0.105–0.515) or lansoprazole (OR 0.441; 95%CI 0.252–0.771). However, there was no significant difference between HetEMs and PMs taking either one. No significant differences were observed for rabeprazole or esomeprazole across the *CYP2C19* genotypes of interest.

**Conclusions:**

Carriage of *CYP2C19* loss-of-function variants is associated with increased *H. pylori* eradication rate in patients taking PPI-based triple therapies when omeprazole or lansoprazole is chosen. However, there is no a class effect after use of rabeprazole or esomeprazole.

## Introduction

It has been well indicated that *Helicobacter pylori* (also known as *H. pylori*) infection is the major risk factor for developing chronic gastritis and peptic ulcer, and is also associated with gastric mucosa-associated lymphoid tissue lymphoma and gastric cancer [1]–[5]. Eradication of *H. pylori* infection is recommended to reduce the recurrence of such diseases [6]–[7]. Current therapy regimens used for the eradication of *H. pylori* are concomitant use of a proton pump inhibitor (PPI) and two antibacterial agents, leading to an eradication rate of 80–90% [8]–[9]. Except for the antisecretory property, PPI can also enhance the efficacy of the antibiotics through decreased antibiotic decay within the gastric juice and increased sensitivity of *H. pylori* to antibiotics [10]–[11]. At present, several PPIs are mainly marketed for patient care, such as omeprazole, esomeprazole (i.e., the pure *S*-isomer of omeprazole), lansoprazole, pantoprazole, and rabeprazole. Accumulating evidence has shown that PPIs are mainly metabolized by the cytochrome P450 (CYP) enzymes (in particular CYP2C19) [12]–[13], and that the phenotype of CYP2C19 is categorized into three groups: extensive metabolizer (EM), intermediate metabolizer (IM), and poor metabolizer (PM). Furthermore, the homozygous EM (HomEM) harbors 2 wild-type alleles (or *1/*1), heterozygous EM (HetEM) carries 1 loss-of-function (LOF) variant allele (frequently *2 or *3), and PM has 2 LOF variant alleles (*2/*2, or *2/*3) [14]–[15]. Because most PPIs are the substrates for CYP2C19, and thus carriers of the *CYP2C19* LOF variants would have increased plasma drug concentrations due to impaired drug metabolism in the liver [15], with an exception of rabeprazole whose metabolism is partly CYP2C19-mediated [13], [16]. Many clinical trials have been published concerning effect of the *CYP2C19* genotypes on the eradication of *H. pylori* by PPI-based triple therapies [17]–[32]. However, there were conflicting conclusions obtained from the currently available randomized clinical trial (RCT) datasets. Moreover, three meta-analysis studies derived from the RCTs or cohort studies were conducted to evaluate the impact of *CYP2C19* variants on the eradication of *H. pylori* in patients treated with PPI-based therapy, but there was less consistency across them [33]–[35]. In addition, few data were available about pantoprazole and esomeprazole in these meta-analysis studies [33]–[35]. Although several trials have determined the effect of *CYP2C19* genotypes on the efficacy of esomeprazole-based therapy [26], [29], [31], effect of *CYP2C19* genotypes in most RCTs was not observed. In line of the fact that there may be a class effect in CYP2C19-dependent PPI metabolism and that RCTs are the gold standard to determine the clinical efficacy and outcomes of a drug that goes to the market, it is necessary to systematically summarize and evaluate the influence of *CYP2C19* variants on all PPI-based triple therapy regimens for *H. pylori* eradication, based on the results from RCTs.

## Methods

### Search Strategy

All relevant information was retrieved up to February 2013 by search of the PubMed, EMBASE, the Cochrane Register of Controlled Trials (CENTRAL), ISI Web of Science, and two Chinese databases (CNKI, and Wanfang) for English and Chinese studies that evaluated the effects of *CYP2C19* polymorphism on the eradication of *H.* pylori based on PPI-based triple therapy. The search strategy – [(*Helicobacter* OR *Helicobacter pylori* OR *Helicobacter* infection) AND (proton pump inhibitor OR PPI OR omeprazole OR lansoprazole OR rabeprazole OR esomeprazole OR pantoprazole) AND (CYP2C19 OR cytochrome P450)]– was used [33]. The search was conducted without language restriction. In addition, the references listed in the retrieved articles and reviews were searched manually. For the missing data, the authors were contacted for detailed information. This meta-analysis was conducted and reported according to the checklists of Preferred Reporting Items for Systematic Reviews and Meta-Analyses (PRISMA) [36].

### Study Selection

For the meta-analysis, all articles had to meet the following inclusion criteria: (1) at least one arm of triple PPI-based therapy for 7–14 days; (2) patients positive for *H. pylori* infection prior to treatment; (3) patients naïve to therapy and genotyped for *CYP2C19*, such as PM, HomEM, and HetEM; and (4) RCTs. Duplicate publications or studies published only in abstract were excluded. Risk of bias of the RCTs was assessed as “low”, “unclear or “high” according to the Cochrane risk of bias tool by the following dominions: randomization method, allocation concealment, blinding, incomplete outcome data addressed and selective reporting [37]. According to the above criteria, two reviewers (H-LT and YL) evaluated the article eligibility independently. The final inclusion decision was made based on the pre-specified consensus among the reviewers or consultation with a third reviewer (S-DZ).

### Data Management

Data from all eligible articles were extracted independently by the two reviews (H-LT and YL), and discrepancy in data extraction was solved through the consensus or consulted with a third reviewer (S-DZ). A standardized data extraction form was designed to extract the major items of information, including author (year), basic characteristics of patients, treatment regimen and the eradication rate of *H. pylori* for PM, HetEM, and HomEM, respectively. In addition, clinical efficacy from the different treatment regimens was also presented in that form.

### Statistical Analysis

Meta-analysis was performed for each PPI regimen alone or in combination according to the genotype. A sub-analysis for individual PPIs associated with the genotype was undertaken to calculate odds ratio (OR) and 95% confidence intervals (CIs). Heterogeneity test was carried out for each combined analysis, where *p<*0.1 indicated significant heterogeneity across the studies. If heterogeneity was insignificant, data from individual studies were pooled using the fixed-effects model. However, data were also combined by using a random-effects model as a sensitivity analysis to confirm the estimated effect due to the therapies given and the difference in populations across the studies. Funnel plot was used to evaluate publication bias if there were more than ten studies included [38], and bias of each group for the endpoints was not found. All statistics was performed with STATA 10.0 (Lakeway Drive College Station, Texas, USA).

## Results

### Characteristics of the Articles Included in the Meta-analysis

**Figure 1:**
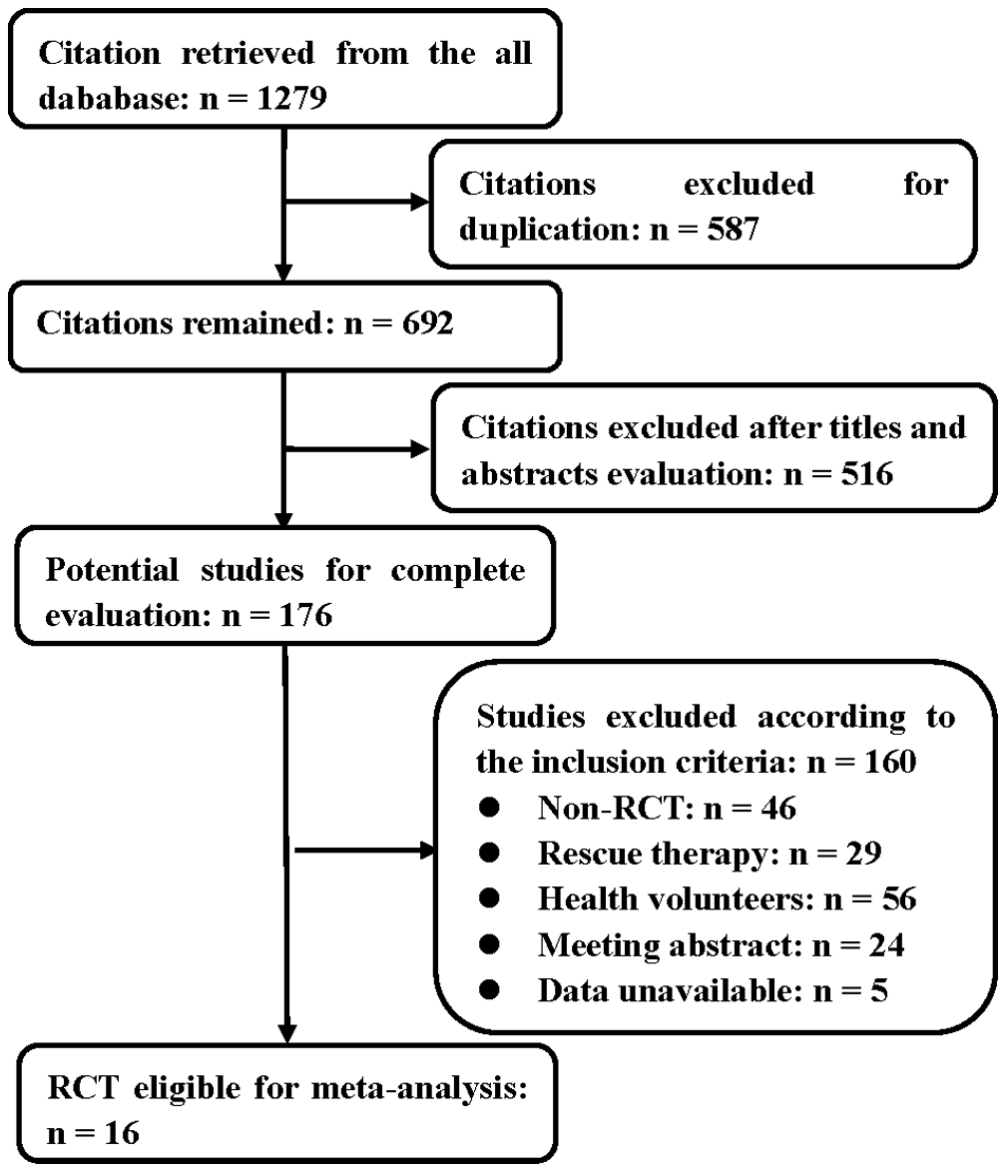
Process that identified eligible randomized clinical trials.

Overall, 16 RCTs of 1279 citations were considered to meet the inclusion criteria, and included in the meta-analysis (Figure 1). Of them, the number of esomeprazole arm, lansoprazole arm, omeprazole arm, rabeprazole arm was 4, 9, 6 and 13, respectively, totaling 3636 patients. Characteristics and the risk of bias of all included studies are summarized in Table 1. One lansoprazole arm was derived from Isomoto et al [19], which compared lansoprazole-based triple therapy with lafutidine-based triple therapy. Various doses of esomeprazole evaluated in the trial by Pan et al [31] were included in the sub-analysis of combined data on esomeprazole. The study by Furuta et al [27] (which evaluated doses of lansoprazole based on the *CYP2C19* genotype) was also included in our meta-analysis. As shown in each trial, no significant difference in the eradication rate was observed among these PPIs.

**Table 1:** Characteristics of the included studies and summary of the eradication rates.

Study	N	Basic character of patients	Treatment regimen	Eradication Rate (n/N)					Risk of bias
				HomEM	HetEM	PM	*CYP2C19* ^a^	Efficacy^b^	
Dojo 2001	170	Japanese *H. pylori*-positive chronic gastritis; M/F:87/83;age: 43±0.6 yr	OAC (20 mg bid);	22/30	31/36	17/20	n/a	No	Unclear
			RAC (20 mg bid )	17/21	34/41	14/16	n/a		
Inaba 2002	183	Japanese *H. pylori*-positive peptic ulcer disease; M/F:142/41;age: 20–83 yr	OAC (20 mg bid);	16/21	24/27	9/10	n/a	No	Low
			RAC (20 mg bid )	18/20	26/29	8/9	Yes		
			LAC (30 mg bid)	15/24	27/31	7/8	n/a		
Isomoto 2003	122	Japanese *H. pylori*-positive peptic ulcer or nonulcer dyspepsia;M/F:90/32; age: 52.1 (21–79) yr	LAC (30 mg bid)	16/21	22/28	11/12	n/a	No	Unclear
			Lafutidine -AC	18/21	27/34	7/9	n/a		
Kawabata 2003	187	Japanese *H. pylori-*positive peptic ulcer disease; M/F:138/49;age: 52 (20–78) yr	RAC (20 mg bid )	26/30	43/53	6/10	Yes	No	Unclear
			LAC (30 mg bid)	24/33	26/35	10/12	Yes		
Miki 2003	145	Japanese *H. pylori*-positive gastritis or peptic ulcers; M/F:113/32,age: 48.9±17.3 yr	RAC (20 mg bid )	11/13	23/26	5/6	n/a	No	High
			RAC (10 mg bid )	18/19	18/20	5/7	n/a		
			LAC (30 mg bid)	10/12	22/26	7/9	n/a		
Take 2003	249	Japanese *H. pylori*-positive peptic ulcer disease; M/F: 219/30;age: 48.7±7.2 yr	OAC (20 mg bid);	56/72	93/119	36/40	n/a	No	High
			RAC (20 mg bid )						
			LAC (30 mg bid)						
Okudaira 2005	177	Japanese *H. pylori*-positive gastritis or peptic ulcersage; age: 44 yr	LACFa (30 mg bid)	29/34	35/41	11/11	n/a	Yes	High
			LAC (30 mg bid)	22/35	40/46	6/6	Yes		
Furuta 2007	300	Japanese *H. pylori*-positive gastritis or peptic ulcers; M/F:192/108;age: 60 (17–89) yr	LAC (*CYP2C19*- based)	54/54	69/73	21/23	n/a	Yes	Low
			LAC (30 mg bid)	30/52	53/74	22/24	Yes		
Kuwayama 2007	479	Japanese *H. pylori*-positive gastritis or peptic ulcers; M/F:331/128;age: 50 yr	RAC (10 mg bid )	33/39	53/63	16/17	n/a	No	Unclear
			RAC (10 mg bid )	28/32	51/58	18/19			
			RAC (20 mg bid )	30/36	56/60	20/20			
			RAC (20 mg bid )	37/42	44/49	23/24			
He 2004	128	Chinese *H. pylori*-positive gastritis or peptic ulcers; M/F:68/60;age: 48.3±15.6 yr	RAC (10 mg bid )	28/35	45/57	19/23	n/a	Yes	High
			RAM (10 mg bid)						
Jiang 2004	169	Chinese *H. pylori*-positive gastritis; M/F:101/68; age: 41 yr	RAC (10 mg bid )	19/23	38/45	10/10	n/a	No	High
			OAC (20 mg bid)	17/30	35/40	11/12	Yes		
Sheu 2005	200	Chinese *H. pylori*-positive gastritis or peptic ulcers; M/F:101/99;age: 41 yr	OAC (20 mg bid)	31/41	27/30	21/22	Yes	No	Unclear
			EAC (40 mg bid)	39/42	28/30	19/20	n/a		
Zhang 2009	240	Chinese *H. pylori*-positive gastritis or peptic ulcers; M/F:181/59;age: 18–70 yr	OAC (20 mg bid)	28/36	49/54	18/19	n/a	No	High
			EAC (40 mg bid)	34/35	47/55	25/27	n/a		
Pan 2010	184	Chinese *H. pylori*–positive gastritis or peptic ulcers; M/F:85/99; age: 44 yr	RAC (10 mg bid )	16/19	13/21	11/14	n/a	No	Unclear
			EALe (40 mg bid)	11/15	18/19	12/12	n/a		
			EALe (20 mg bid)	14/16	19/21	9/10	n/a		
Zhang 2010	240	Chinese *H. pylori*-positive peptic ulcer disease; M/F:194/46; age: 45.2±14.2 yr	RAC (10 mg bid )	33/34	50/59	20/22	n/a	No	High
			OAC (20 mg bid)	28/36	49/54	18/19	n/a		
Lee 2010	463	Korean *H. pylori*-positive peptic ulcer disease, post-endoscopic mucosal resection of gastric cancer or adenoma, not ulcer dyspepsia; M/F:276/187; age: 56 yr	RAC (10 mg bid )	59/86	81/111	23/32	n/a	No	High
			LAC (30 mg bid)	63/85	87/108	35/41	n/a		

O, omeprazole; L, lansoprazole; R, rabeprazole; E, esomeprazole, A, amoxicillin; C, clarithromycin; M, metronidazole; Fa, famotidine; Le, levofloxacin; HomEM, homozygous extensive metabolizers; HetEM, heterozygous extensive metabolizers; PM, poor metabolizer; ITT, intent-to-treat; M, male; F, female. Year, yr; n/a, not available, ^a^ effect by the *CYP2C19* genotype; ^b^ effect between randomized groups.

### Effects of *CYP2C19* Genotypes on the Overall Efficacy of All PPI-based Triple Therapies

**Figure 2:**
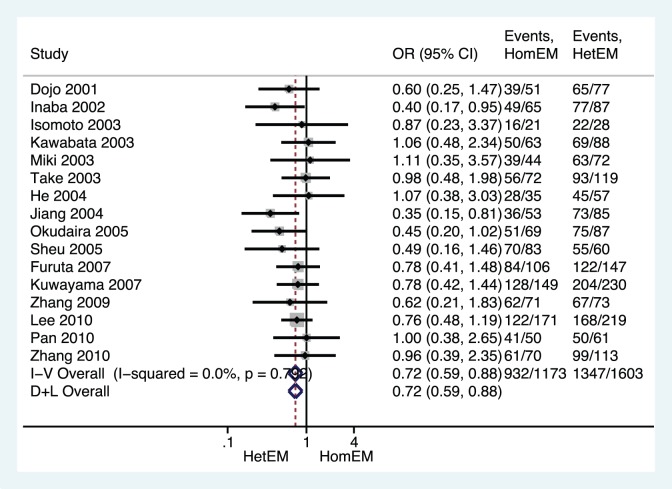
Forest plot of RCTs comparing HomEMs vs. **HetEM in relation to ***H. pylori* eradication rate of all PPI-based triple therapies.****

**Figure 3:**
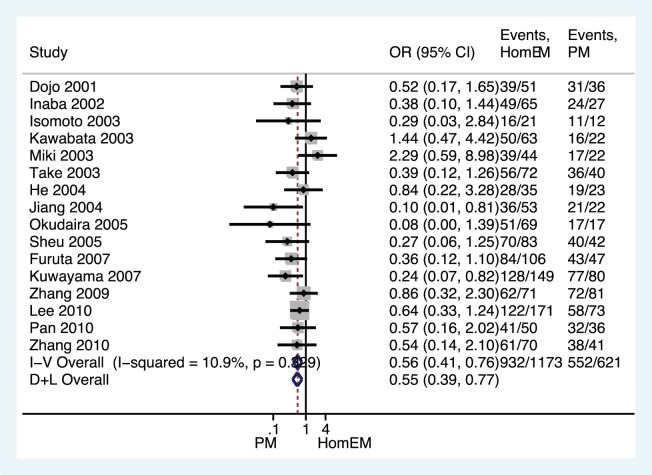
Forest plot of RCTs comparing HomEMs vs. **PM in relation to ***H. pylori* eradication rate of all PPI-based triple therapies.****

**Figure 4:**
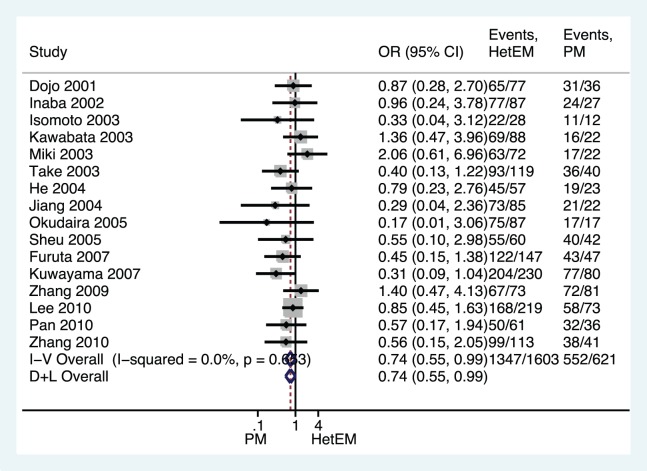
Forest plot of RCTs comparing HetEMs vs. **PM in relation to ***H. pylori* eradication rate of all PPI-based triple therapies.****

All PPI-based triple therapies, regardless of the doses and antibiotics used, were combined in our initial analysis, and a significant difference in *H. pylori* eradication rates was found between the HomEM and HetEM genotype groups as shown in Figure 2 (OR 0.724, 95%CI 0.594–0.881; *p = *0.001), between the HomEM and PM genotype groups (Figure 3, OR 0.507, 95%CI 0.379–0.679; *p*<0.001), or between the HetEM and PM genotype groups (Figure 4, OR 0.688, 95%CI 0.515–0.920; *p = *0.012) with the fixed-effects model, due to no significant heterogeneity across all the studies (all *p*>0.1). In addition, by using a random effects model, a sensitivity analysis showed that results were robust as showed in Figures 2–4.

### Effects of *CYP2C19* Genotypes on the Efficacy of Individual PPI-based Triple Therapies

Because there was no significant heterogeneity across all the studies (all *p*>0.1), a fixed effects model was used firstly. Results of the meta-analysis showed that a significant difference existed in the *H. pylori* eradication rates with omeprazole-based triple therapy between the HomEM and PM genotypes (OR 0.232, 95%CI 0.105–0.515; *p*<0.0001), between HomEM and HetEM genotypes (OR 0.329, 95%CI 0.195–0.533; *p*<0.001), but not between the HetEM and PM genotypes (OR 0.694, 95%CI 0.299–1.608, *p = *0.394). Similarly, lansoprazole-based triple therapy exhibited a significant difference in the *H. pylori* eradication rates between HomEM and PM genotypes (OR 0.441, 95%CI 0.252–0.771; *p = *0.004), between HomEM and HetEM genotypes (OR 0.692, 95%CI 0.485–0.988, *p = *0.043), but not between HetEM and PM genotypes (OR 0.584, 95%CI 0.333–1.024; *p = *0.778). Contrary to the above, rabeprazole- or esomeprazole-based triple therapies did not show any significant difference in *H. pylori* eradication rates among all three genotypes (Table 2). Furthermore, results of the sub-analysis by a random effects model were in consistency with above results.

**Table 2:** Effect of *CYP2C19* genotypes on the eradication rate of individual PPI-based triple therapies.

Subgroup	HomEM *vs.* HetEM			HomEM *vs.* PM				HetEM *vs.* PM		
	N	OR (95%CI); sig	Het		N	OR (95%CI); sig	Het	N	OR (95%CI); sig	Het
Omeprazole	435	0.329 (0.195–0.553), *p* = 0.000	*P = *0.945		296	0.232 (0.105–0.515), *p<*0.001	*P = *0.890	343	0.694 (0.299–1.608), *p* = 0.394	*P = *0.986
Lansoprazole	806	0.692 (0.485–0.988), *p* = 0.043	*P = *0.540		493	0.441 (0.252–0.771), *p* = 0.004	*P = *0.275	607	0.584 (0.333–1.024), *p* = 0.060	*P = *0.778
Esomeprazole	232	1.000 (0.410–2.437), *p = *0.999	*P = *0.185		177	0.623 (0.200–1.942), *p* = 0.414	*P = *0.426	193	0.642 (0.209–1.975), *p* = 0.440	*P = *0.973
Rabeprazole	1055	0.931 (0.669–1.294), *p* = 0.669	*P = *0.396		623	0.769 (0.477–1.238), *p* = 0.279	*P = *0.303	842	0.778 (0.491–1.232), *p* = 0.284	*P = *0.716

Data were presented with odds ratio (OR) and 95% CI; sig, significance; Het, heterogeneity test.

## Discussion

The results about the effects of *CYP2C19* variants on the *H. pylori* eradication rate in patients treated with PPI-based triple therapies were controversial across all relevant clinical trials and three published meta-analyses [33]–[35]. Our findings clearly confirmed that clinical efficacy of PPI-based triple therapies on the eradication rate was influenced by *CYP2C19* genotype status. There was a significant difference in the eradication rate among the three genotypes for some but not all PPI-based triple therapy regimens, such as omeprazole, and lansoprazole. As expected, a higher eradication rate in PMs than in HomEM, in PMs than in HetEM, and in HetEM than in HomEM were observed, suggesting a gene dosage effect on the metabolism of PPIs and also on the eradication of *H. pylori*, consistent with the conclusions reported by a previously published meta-analysis [34].

Considering the difference among individual PPI-based triple therapies influenced by the *CYP2C19* genotype, sub-analysis of individual PPIs was conducted to explore the effects of *CYP2C19* genotypes on each PPI. Significant differences were observed on the *H. pylori* eradication between HomEM and HetEM, between HomEM and PM, but not between HetEM and PM genotypes in patients taking lansoprazole- or omeprazole-based triple therapies. This result may be explained, at least in part, by the observation that omeprazole is a mechanism-based inhibitor of CYP2C19 [39]. The antisecretory activity of omeprazole or lansoprazole is expected to be different between the genotypes of *CYP2C19*, and thus, the cure rate of *H. pylori* of omeprazole- or lansoprazole-based regimens should be influenced by the *CYP2C19* genotype. Our study showed that patients with PM genotype had a higher cure rate of *H. pylori* than those with HomEM genotype by about 80% and 60% in those taking omeprazole and lansoprazole, respectively, suggesting that patients with HomEM genotype may need to take a higher-than-standard dose of omeprazole or lansoprazole. The meta-analysis by McNicholl et al. showed that non-CYP2C19 metabolized PPIs (such as rabeprazole or esomeprazole) could achieve a higher cure rate of *H. pylori* in patients with EMs than CYP2C19 metabolized PPIs (such as omeprazole or lansoprazole) [35]. Therefore, it is necessary to use higher dose of omeprazole or lansoprazole or non-CYP2C19-metabolized PPIs such as rabeprazole, in order to minimize or even avoid the effect of *CYP2C19* genotypes.

Similar to most studies, no significant association was observed between *CYP2C19* genotypes and *H. pylori* eradication rates in patients taking rabeprazole- or esomeprazole-based triple therapies [29], [40]. Previous studies have confirmed that esomeprazlole is metabolized, to a less content, by CYP2C19 than omeprazole [41], because esomeprazole is a pure *S*-isomer of omeprazole, which is different from omeprazole composed of the equal amount of *R*- and *S*-isomer. The proportion of the *S*-isomer metabolized via CYP2C19 is less than that of the *R*-isomer, resulting in less interindividual variation in plasma drug concentrations than omeprazole [42]. Our meta-analysis results suggest that esomeprazole is not significantly affected by the *CYP2C19* genotype, and that influence of small sample sizes in individual studies seems to be less important as expected. Similarly, rabeprazole is metabolized to thioether-rabeprazole mainly via a non-enzymatic pathway, with minor involvement of CYP2C19 [43], consistent with the results derived from our meta-analysis. Thus, esomeprazole- or rabeprazole-based triple therapies with the standard dose can be used to eradicate *H. pylori* infection for all patients, without need in considering the status of *CYP2C19* polymorphisms. However, in terms of the fact that the sample size of most clinical investigations was small, it was not observed that carriage of different *CYP2C19* genotypes is not associated with the eradication rate of the *H. pylori* infection in patients treated with PPI-based triple therapies. Our meta-analysis overcame the limitation of power by pooling such studies. Except for the *CYP2C19* genotype, antibiotic resistance, and interleukin (IL)-1ß genotype may play an important role in affecting the eradication rates. For example, when nitroimidazole/clarithromycin resistance was occurred, the *H. pylori* eradication rate dropped significantly in patients treated with most PPI-based triple therapy regimens [44]–[45]. IL-1ß genotype was considered to influence the cure rate of PPI-based eradication therapy for *H. pylori*, by affecting gastric acid secretion [46]–[47]. However, effect of IL-1ß genotype on *H. pylori* eradication was still controversial [22], [32], [48]–[49]. It is necessary to evaluate the effect of these factors on the *H. pylori* eradication by PPI-based therapy.

Our results were in agreement with that from meta-analysis of the observational trials by Zhao et al [34]. However, effect of the *CYP2C19* genotype on pantoprazole is not evaluated in our meta-analysis, due to limited randomized trials available. A non-randomized trial showed that *CYP2C19* genotype might affect the efficacy of *H. pylori* eradication in peptic ulcer patients treated with pantoprazole [49]. Different from above-mentioned meta-analysis performed for clinical observational studies [33]–[34], only RCTs were included in our meta-analysis, because RCTs can minimize the influence of various confounding factors or bias on clinical efficacy of that therapy strategy for that indication. However, in our meta-analysis, individual PPIs were pooled without considering the dose, duration of therapy and the type of antibiotic agents, resulting in some confounders for *CYP2C19* phenotypes and the eradication rates of PPI-based therapy. Therefore, it may not be extended well to clinical practice. However, no significant heterogeneity and publication bias were found in our meta-analysis, suggesting that our conclusions seem to be reasonable.

In summary, the *CYP2C19* variant carriage is the major determinant of altered *H. pylori* eradication rate in patients taking PPI-based triple therapies when omeprazole or lansoprazole is chosen. In contrast, the *CYP2C19* polymorphism has less effect on that eradication rate after use of rabeprazole or esomeprazole. Choice of different PPIs and/or doses should be individualized based on the pharmacogenetics background of each patient and pharmacological profile of each drug in the human body.

## References

[1] Marshall BJ, Warren JR (1984) Unidentified curved bacilli in the stomach of patients with gastritis and peptic ulceration. Lancet 1: 1311–1315. 6145023 10.1016/s0140-6736(84)91816-6

[2] NIH Consensus Conference. Helicobacter pylori in peptic ulcer disease. NIH Consensus Development Panel on Helicobacter pylori in Peptic Ulcer Disease. JAMA 272: 65–69. 8007082

[3] Goodwin CS, Mendall MM, Northfield TC (1997) Helicobacter pylori infection. Lancet 349: 265–269. 9014926 10.1016/S0140-6736(96)07023-7

[4] Uemura N, Okamoto S, Yamamoto S, Matsumura N, Yamaguchi S, et al (2001) Helicobacter pylori infection and the development of gastric cancer. N Engl J Med 345: 784–789. 11556297 10.1056/NEJMoa001999

[5] Marshall BJ, Windsor HM (2005) The relation of Helicobacter pylori to gastric adenocarcinoma and lymphoma: pathophysiology, epidemiology, screening, clinical presentation, treatment, and prevention. Med Clin North Am 89: 313–344, viii. 10.1016/j.mcna.2004.09.001 15656929

[6] Marshall BJ, Goodwin CS, Warren JR, Murray R, Blincow ED, et al (1988) Prospective double-blind trial of duodenal ulcer relapse after eradication of Campylobacter pylori. Lancet 2: 1437–1442. 2904568 10.1016/s0140-6736(88)90929-4

[7] Chey WD, Wong BC (2007) American College of Gastroenterology guideline on the management of Helicobacter pylori infection. Am J Gastroenterol 102: 1808–1825. 17608775 10.1111/j.1572-0241.2007.01393.x

[8] Egan BJ, Katicic M, O’Connor HJ, O’Morain CA (2007) Treatment of Helicobacter pylori. Helicobacter 12 Suppl 131–37. 10.1111/j.1523-5378.2007.00538.x 17727458

[9] Malfertheiner P, Megraud F, O’Morain C, Bazzoli F, El-Omar E, et al (2007) Current concepts in the management of Helicobacter pylori infection: the Maastricht III Consensus Report. Gut 56: 772–781. 17170018 10.1136/gut.2006.101634 PMC1954853

[10] Goddard AF, Jessa MJ, Barrett DA, Shaw PN, Idstrom JP, et al (1996) Effect of omeprazole on the distribution of metronidazole, amoxicillin, and clarithromycin in human gastric juice. Gastroenterology 111: 358–367. 8690200 10.1053/gast.1996.v111.pm8690200

[11] Erah PO, Goddard AF, Barrett DA, Shaw PN, Spiller RC (1997) The stability of amoxycillin, clarithromycin and metronidazole in gastric juice: relevance to the treatment of Helicobacter pylori infection. J Antimicrob Chemother 39: 5–12. 9044021 10.1093/jac/39.1.5

[12] Andersson T, Regardh CG, Dahl-Puustinen ML, Bertilsson L (1990) Slow omeprazole metabolizers are also poor S-mephenytoin hydroxylators. Ther Drug Monit 12: 415–416. 2396316 10.1097/00007691-199007000-00020

[13] Ishizaki T, Horai Y (1999) Review article: cytochrome P450 and the metabolism of proton pump inhibitors–emphasis on rabeprazole. Aliment Pharmacol Ther 13 Suppl 327–36. 10.1046/j.1365-2036.1999.00022.x 10491726

[14] Tomalik-Scharte D, Lazar A, Fuhr U, Kirchheiner J (2008) The clinical role of genetic polymorphisms in drug-metabolizing enzymes. Pharmacogenomics J 8: 4–15. 17549068 10.1038/sj.tpj.6500462

[15] Hagymasi K, Muellner K, Herszenyi L, Tulassay Z (2011) Update on the pharmacogenomics of proton pump inhibitors. Pharmacogenomics 12: 873–888. 21692617 10.2217/pgs.11.4

[16] Dadabhai A, Friedenberg FK (2009) Rabeprazole: a pharmacologic and clinical review for acid-related disorders. Expert Opin Drug Saf 8: 119–126. 19236223 10.1517/14740330802622892

[17] Dojo M, Azuma T, Saito T, Ohtani M, Muramatsu A, et al (2001) Effects of CYP2C19 gene polymorphism on cure rates for Helicobacter pylori infection by triple therapy with proton pump inhibitor (omeprazole or rabeprazole), amoxycillin and clarithromycin in Japan. Dig Liver Dis 33: 671–675. 11785712 10.1016/s1590-8658(01)80043-8

[18] Inaba T, Mizuno M, Kawai K, Yokota K, Oguma K, et al (2002) Randomized open trial for comparison of proton pump inhibitors in triple therapy for Helicobacter pylori infection in relation to CYP2C19 genotype. J Gastroenterol Hepatol 17: 748–753. 12121503 10.1046/j.1440-1746.2002.02790.x

[19] Isomoto H, Inoue K, Furusu H, Nishiyama H, Shikuwa S, et al (2003) Lafutidine, a novel histamine H2-receptor antagonist, vs lansoprazole in combination with amoxicillin and clarithromycin for eradication of Helicobacter pylori. Helicobacter 8: 111–119. 12662378 10.1046/j.1523-5378.2003.00131.x

[20] Kawabata H, Habu Y, Tomioka H, Kutsumi H, Kobayashi M, et al (2003) Effect of different proton pump inhibitors, differences in CYP2C19 genotype and antibiotic resistance on the eradication rate of Helicobacter pylori infection by a 1-week regimen of proton pump inhibitor, amoxicillin and clarithromycin. Aliment Pharmacol Ther 17: 259–264. 12534411 10.1046/j.1365-2036.2003.01406.x

[21] Miki I, Aoyama N, Sakai T, Shirasaka D, Wambura CM, et al (2003) Impact of clarithromycin resistance and CYP2C19 genetic polymorphism on treatment efficacy of Helicobacter pylori infection with lansoprazole- or rabeprazole-based triple therapy in Japan. Eur J Gastroenterol Hepatol 15: 27–33. 12544691 10.1097/00042737-200301000-00006

[22] Take S, Mizuno M, Ishiki K, Nagahara Y, Yoshida T, et al (2003) Interleukin-1beta genetic polymorphism influences the effect of cytochrome P 2C19 genotype on the cure rate of 1-week triple therapy for Helicobacter pylori infection. Am J Gastroenterol 98: 2403–2408. 14638340 10.1111/j.1572-0241.2003.07707.x

[23] He XX, Zhao YH, Hao YT (2004) Effect of CYP2C19 genetic polymorphism on treatment efficacy of Helicobacter pylori infection with rabeprazole-based triple therapy in Chinese. Chin J Intern Med 43: 13–15. 14990013

[24] Jiang YJ, Li YY, Nie YQ, Wang H, Sha WH (2004) Effect of Rabeprazole on Eradication of Helicobacter Pylori and Its Correlation to CYP2C19 Genetic Polymorphisms. Academic Journal of Guangzhou Medical College 32: 22–25.

[25] Okudaira K, Furuta T, Shirai N, Sugimoto M, Miura S (2005) Concomitant dosing of famotidine with a triple therapy increases the cure rates of Helicobacter pylori infections in patients with the homozygous extensive metabolizer genotype of CYP2C19. Aliment Pharmacol Ther 21: 491–497. 15710002 10.1111/j.1365-2036.2005.02353.x

[26] Sheu BS, Kao AW, Cheng HC, Hunag SF, Chen TW, et al (2005) Esomeprazole 40 mg twice daily in triple therapy and the efficacy of Helicobacter pylori eradication related to CYP2C19 metabolism. Aliment Pharmacol Ther 21: 283–288. 15691303 10.1111/j.1365-2036.2005.02281.x

[27] Furuta T, Shirai N, Kodaira M, Sugimoto M, Nogaki A, et al (2007) Pharmacogenomics-based tailored versus standard therapeutic regimen for eradication of H. pylori. Clin Pharmacol Ther 81: 521–528. 17215846 10.1038/sj.clpt.6100043

[28] Kuwayama H, Asaka K, Sugiyama T, Fukuda Y, Aoyama N, et al (2007) Rabeprazole-based eradication therapy for Helicobacter pylori: a large-scale study in Japan. Aliment Pharmacol Ther 25: 1105–1113. 17439512 10.1111/j.1365-2036.2007.03298.x

[29] Zhang L, Xu JM, Mei Q, Li QS, Hu YM (2009) Impact of CYP2C19 polymorphisms on eradication of Helicobacter pylori using triple therapy with esomeprazole. Chin J Dig 29: 545–548.

[30] Lee JH, Jung HY, Choi KD, Song HJ, Lee GH, et al (2010) The Influence of CYP2C19 Polymorphism on Eradication of Helicobacter pylori: A Prospective Randomized Study of Lansoprazole and Rabeprazole. Gut Liver 4: 201–206. 20559522 10.5009/gnl.2010.4.2.201 PMC2886925

[31] Pan X, Li Y, Qiu Y, Tang Q, Qian B, et al (2010) Efficacy and tolerability of first-line triple therapy with levofloxacin and amoxicillin plus esomeprazole or rabeprazole for the eradication of Helicobacter pylori infection and the effect of CYP2C19 genotype: a 1-week, randomized, open-label study in Chinese adults. Clin Ther 32: 2003–2011. 21118735 10.1016/j.clinthera.2010.11.005

[32] Zhang L, Mei Q, Li QS, Hu YM, Xu JM (2010) The effect of cytochrome P2C19 and interleukin-1 polymorphisms on H. pylori eradication rate of 1-week triple therapy with omeprazole or rabeprazole, amoxycillin and clarithromycin in Chinese people. J Clin Pharm Ther 35: 713–722. 21054464 10.1111/j.1365-2710.2009.01140.x

[33] Padol S, Yuan YH, Thabane M, Padol IT, Hunt RH (2006) The effect of CYP2C19 polymorphisms on H-Pylori eradication rate in dual and triple first-line PPI therapies: A meta-analysis. Am J Gastroenterol 101: 1467–1475. 16863547 10.1111/j.1572-0241.2006.00717.x

[34] Zhao F, Wang J, Yang Y, Wang X, Shi R, et al (2008) Effect of CYP2C19 Genetic Polymorphisms on the Efficacy of Proton Pump Inhibitor-Based Triple Therapy for Helicobacter pylori Eradication: A Meta-Analysis. Helicobacter 13: 532–541. 19166419 10.1111/j.1523-5378.2008.00643.x

[35] McNicholl AG, Linares PM, Nyssen OP, Calvet X, Gisbert JP (2012) Meta-analysis: esomeprazole or rabeprazole vs. first-generation pump inhibitors in the treatment of Helicobacter pylori infection. Aliment Pharmacol Ther 36: 414–425. 22803691 10.1111/j.1365-2036.2012.05211.x

[36] Moher D, Liberati A, Tetzlaff J, Altman DG (2009) Preferred reporting items for systematic reviews and meta-analyses: the PRISMA statement. PLoS Med 6: e1000097. 19621072 10.1371/journal.pmed.1000097 PMC2707599

[37] Higgins JPT, Green S (2011) Cochrane Handbook for Systematic Reviews of Interventions Version 5.1.0 [updated March 2011]. Available: http://handbook.cochrane.org. Accessed 15 December 2012.

[38] Ioannidis JP, Trikalinos TA (2007) The appropriateness of asymmetry tests for publication bias in meta-analyses: a large survey. CMAJ 176: 1091–1096. 17420491 10.1503/cmaj.060410 PMC1839799

[39] Furuta T, Ohashi K, Kosuge K, Zhao XJ, Takashima M, et al (1999) CYP2C19 genotype status and effect of omeprazole on intragastric pH in humans. Clin Pharmacol Ther 65: 552–561. 10340921 10.1016/S0009-9236(99)70075-5

[40] Hokari K, Sugiyama T, Kato M, Saito M, Miyagishima T, et al (2001) Efficacy of triple therapy with rabeprazole for Helicobacter pylori infection and CYP2C19 genetic polymorphism. Aliment Pharmacol Ther 15: 1479–1484. 11552922 10.1046/j.1365-2036.2001.01063.x

[41] Dent J (2003) Review article: pharmacology of esomeprazole and comparisons with omeprazole. Aliment Pharmacol Ther 17 Suppl 15–9. 12614299 10.1046/j.1365-2036.17.s1.2.x

[42] Tybring G, Bottiger Y, Widen J, Bertilsson L (1997) Enantioselective hydroxylation of omeprazole catalyzed by CYP2C19 in Swedish white subjects. Clin Pharmacol Ther 62: 129–137. 9284848 10.1016/S0009-9236(97)90060-6

[43] Lim PW, Goh KL, Wong BC (2005) CYP2C19 genotype and the PPIs–focus on rabeprazole. J Gastroenterol Hepatol 20 Suppl: S22–28 10.1111/j.1440-1746.2005.04167.x 16359346

[44] Houben MH, van de Beek D, Hensen EF, de Craen AJ, Rauws EA, et al (1999) A systematic review of Helicobacter pylori eradication therapy–the impact of antimicrobial resistance on eradication rates. Aliment Pharmacol Ther 13: 1047–1055. 10468680 10.1046/j.1365-2036.1999.00555.x

[45] Miwa H, Misawa H, Yamada T, Nagahara A, Ohtaka K, et al (2001) Clarithromycin resistance, but not CYP2C-19 polymorphism, has a major impact on treatment success in 7-day treatment regimen for cure of H. pylori infection: a multiple logistic regression analysis. Dig Dis Sci 46: 2445–2450. 11713950 10.1023/a:1012371702918

[46] Jung HC, Kim JM, Song IS, Kim CY (1997) Helicobacter pylori induces an array of pro-inflammatory cytokines in human gastric epithelial cells: quantification of mRNA for interleukin-8, -1 alpha/beta, granulocyte-macrophage colony-stimulating factor, monocyte chemoattractant protein-1 and tumour necrosis factor-alpha. J Gastroenterol Hepatol 12: 473–480. 9257236 10.1111/j.1440-1746.1997.tb00469.x

[47] El-Omar EM, Carrington M, Chow WH, McColl KE, Bream JH, et al (2000) Interleukin-1 polymorphisms associated with increased risk of gastric cancer. Nature 404: 398–402. 10746728 10.1038/35006081

[48] Mizuno M, Take S, Ishiki K, Okada H, Shiratori Y (2004) Interluekin-1 beta genetic polymorphism influences the impact of cytochrome P 2C19 genotype on the cure rate of H. pylori eradication therapy. Nihon rinsho 62: 455–458. 15038086

[49] Gawronska-Szklarz B, Siuda A, Kurzawski M, Bielicki D, Marlicz W, et al (2010) Effects of CYP2C19, MDR1, and interleukin 1-B gene variants on the eradication rate of Helicobacter pylori infection by triple therapy with pantoprazole, amoxicillin, and metronidazole. Eur J Clin Pharmacol 66: 681–687. 20376628 10.1007/s00228-010-0818-1

